# Whooping-Cough

**Published:** 1875-03

**Authors:** 


Hooping-Cough.—Wilde claims that he can cure every case of hooping-cough within eight days by the following treatment: The patient is not to leave the room, and at every access of coughing is to hold before his mouth a small piece of cloth, folded several times, and with a teaspoonful of this solution: ether, 60 parts; chloroform, 30 parts; turpentine, 10 parts.—*Medical Record, 9th Jan.*, 1875, *fr. Allg. Wien Med. Ztg.*

